# Turning 21: Induction of miR-21 as a Key Switch in the Inflammatory Response

**DOI:** 10.3389/fimmu.2015.00019

**Published:** 2015-01-29

**Authors:** Frederick J. Sheedy

**Affiliations:** ^1^TB Immunology Laboratory, Department of Clinical Medicine, Institute of Molecular Medicine, Trinity College Dublin, Dublin, Ireland

**Keywords:** miR-21, inflammation, cancer, PDCD4, macrophage

## Abstract

miR-21 is one of the most highly expressed members of the small non-coding microRNA family in many mammalian cell types. Its expression is further enhanced in many diseased states including solid tumors, cardiac injury, and inflamed tissue. While the induction of miR-21 by inflammatory stimuli cells has been well documented in both hematopoietic cells of the immune system (particularly monocytes/macrophages but also dendritic and T-cells) and non-hematopoietic tumorigenic cells, the exact functional outcome of this elevated miR-21 is less obvious. Recent studies have confirmed a key role for miR-21 in the resolution of inflammation and in negatively regulating the pro-inflammatory response induced by many of the same stimuli that trigger miR-21 induction itself. In particular, miR-21 has emerged as a key mediator of the anti-inflammatory response in macrophages. This suggests that miR-21 inhibition in leukocytes will promote inflammation and may enhance current therapies for defective immune responses such as cancer, mycobacterial vaccines, or Th2-associated allergic inflammation. At the same time, miR-21 has been shown to promote inflammatory mediators in non-hematopoietic cells resulting in neoplastic transformation. This review will focus on functional studies of miR-21 during inflammation, which is complicated by the numerous molecular targets and processes that have emerged as miR-21 sensitive. It may be that the exact functional outcome of miR-21 is determined by multiple features including the cell type affected, the inducing signal, the transcriptomic profile of the cell, which ultimately affect the availability and ability to engage different target mRNAs and bring about its unique responses. Reviewing this data may illustrate that RNA-based oligonucleotide therapies for different diseases based upon miR-21 may have to target the unique and operative miRNA:mRNA interactions’ functionally active in disease.

## Introduction

Micro-RNA-21 (miR-21) is an abundantly expressed microRNA in mammalian cells of multiple types (1–3). Its up-regulation is associated with many cancers, including those derived from both solid tissue (4, 5) and leukemic origin (6–9). The generation of a conditional miR-21 “knock-in” mouse confirmed that it functions as an oncogene with its overexpression resulting in malignant B-cell lymphoma (10). Functional studies performed in epithelia-, hepatocyte-, and glial cell-derived cell lines confirm that miR-21 regulates processes connected to cell growth, migration, and invasion (11–16), providing a mechanism for miR-21-mediated transformation of somatic cells. However, miR-21 is also expressed in hematopoietic cells of the immune system including B/T-cells, monocytes, macrophages, and dendritic cells (DCs). Activation of the immune system is strongly associated with tumor progression but also with surveying, responding to, and eliminating tumors as they arise. How increased miR-21 in these cell types facilitates tumor progression, as well as orchestrating the general immune response to pathogens and autoantigens in inflammatory disease, remains unclear. In this review, I will attempt to highlight some of the key findings on miR-21’s role in immunity and place this in the context of its dysregulation in disease including cancer and inflammation. I present a model of miR-21 as a key switch in immune circuits, controlling the balance between initial pro-inflammatory and later immuno-regulatory, anti-inflammatory responses, – dysregulation of which contributes to pathogenesis of inflammatory diseases including cancer and infection.

## miR-21 Expression and Induction in Hematopoietic Cells

### Immune cell maturation

Initial efforts to profile miRNA expression during hematopoiesis revealed that while miR-21 is moderately expressed in hematopoietic progenitors, its expression increases significantly as various cell types mature to an “active” state, including bone marrow-derived mast-cells (17), neutrophils (18), and activated T-cells of various lineages (19, 20). High miR-21 levels are therefore a marker of immune cell activation, although whether or not this reflects a cause or consequence of activation remained to be determined. It was found that miR-21 expression is RNA polymerase II-dependent and derived from a primary transcript that is both capped and polyadenylated (21). Similar to regular protein-coding mRNAs, miR-21 expression is dynamically regulated by complex signaling pathways and can be enhanced by extracellular signals during immune cell development. The myeloid precursor cell type, the monocyte, which can be differentiated into various mature cells depending upon the extracellular signals received, shows increased expression of miR-21 during activation. This was first demonstrated by the study of Kashashima et al. (22), where TPA (also known as PMA) was used to differentiate monocytes toward macrophages. Since then, studies showing treatment of monocytes with all-trans retinoic acid to generate neutrophils (18), GM-CSF/IL-4 treatment to generate immature DCs (23, 24), and treatment with LPS (a TLR4 specific mimetic of bacterial endotoxin) to generate activated macrophages (25, 26), as well as LPS-mediated B-cell activation (3), all revealed significant up-regulation of miR-21. Table 1 lists these examples alongside many other immunologically relevant examples, but their detailed discussion is beyond the scope of this review. However, taken together, these data confirm that miR-21 serves as an important marker of immune cell activation in multiple contexts.

**Table 1:** **Select examples of miR-21 induction by inflammatory stimuli**.

Signal	Cell type	Result	Transcriptional control	Post-transcriptional regulation	Reference
PMA	Monocyte	Macrophage differentiation	AP-1, NFIB		(22, 27)

Retinoic acid	Monocyte	Neutrophil differentiation			(18)

IL-6	Multiple myeloma		STAT-3		(28)

TGF-β BMP	Vascular smooth muscle	Contractile phenotype	–	p68, SMAD6	(29)

LPS	B-cells, macrophages	IL-10 production	NFκB		(3)(25, 30)

GM-CSF/IL4	Monocyte	MD-DC			(23, 24)

GM-CSF/IL-6, TGF-β	Bone marrow precursors	MDSC			(31)

TGF-β/TNF	Colon carcinoma culture	EMT	+	+	(32)

*MD-DC, monocyte-derived dendritic cells, MDSC, myeloid-derived suppressor cells; EMT, epithelial–mesenchymal transition*.

### Turning the circuit “on” – induction of miR-21 by inflammatory stimuli

Like regular Pol-II-regulated protein-coding mRNAs, which are regulated by a diverse array of signal-specific transcription factors that bind unique sites at the promoter region, miR-21 exhibits diversity in the signals, transcription factors, and proposed binding sites that regulate its expression in diverse contexts. Unlike, regular Pol-II-regulated protein coding-genes and like all miRNAs, miR-21 is subject to an additional layer of post-transcriptional regulation before the mature 20 nt bioactive form is generated. This involves processing of both the precursor and mature duplex miRNA from the primary miRNA transcript (pri-miR-21), carried out by the nuclear enzyme Drosha and its cytosolic counterpart, Dicer. Many of the induction studies of miR-21 by extracellular signals including TPA/PMA, LPS, IL-6, and TGF-β/BMP have shown it to be a later event in their respective signaling pathways (25, 27–29). Expression analysis downstream of oncogene Ras-induced signaling, which drives miR-21 through AP-1, has shown that the appearance of mature bioactive miR-21 is delayed relative to the generation of pri-miR-21 (33). Although a dearth of studies have defined the role of various transcription factors in the induction of miR-21, including NFκB in LPS-induced miR-21 (25, 30), AP-1 in PMA-mediated up-regulation (27), and STAT-3 for IL-6-induced miR-21 (28), the complexity of the predicted promoter region of pri-miR-21 (27, 28) and the occurrence of alternative transcription start sites (34) suggest that the regulation of miR-21 transcription is not straight forward.

Several studies place miR-21 among the group of miRNAs whose post-transcriptional processing requires extra co-factors, notably the RNA helicase protein p68, which aids cleavage of the pri-miRNA transcript by Drosha (35). Although processing of mature miR-21 relative to other miRs may be differentially regulated by the inherent sequence differences of the miR-21 primary transcript recognized by Drosha/Dicer, a further layer of complexity is added when it is considered that the enzymes involved in miR-21 biogenesis are themselves regulated by extracellular signals, as shown in the study of TGF-β/BMP-mediated induction of mature miR-21. Here SMAD6, a key intracellular adapter protein activated by TGF-β signaling, bound to the primary miR-21 transcript and recruited p68 to promote Drosha-mediated cleavage during TGF-β signalling (29).

Interestingly, in a study of miR-21 induction in a model of colon carcinoma epithelial–mesenchymal transition (EMT), combined treatment with TGF-β and TNF induced pri-miR-21 and at a later stage, the appearance of the Drosha cleavage product, precursor-miR-21 stem-loop (32). This latter event required *de novo* protein synthesis and is indicative of an additional regulatory step to organize the temporal and cell-specific induction of miR-21. This important finding may be applicable to immune cells, which rapidly induce many cytokines and secreted factors, such as IL-6 or TNF, that have the potential to feed back and drive later events in the cell.

The story of miR-21 regulation grows even more complex when we consider the impact of other non-coding RNAs on its expression. A recent study illustrated that miR-21 is in fact regulated by a member of the long non-coding RNA family, GAS5 (36). Although lncRNAs can regulate genes at the transcriptional level, they also hold the potential to act as miRNA sponges – mopping up excessive mature 20 nt miRNAs and preventing them from engaging their mRNA targets. Therefore, GAS5 may in fact represent an important negative regulator of miR-21 activity, although this has yet to be examined in immune cells.

### Expression in diseased tissue

Coincident with its induction in various immune cell types *ex vivo*, *in vivo* studies of diseased tissue often demonstrate increased expression of miR-21 relative to healthy control tissue. This has been shown in various models of allergic airway inflammation (26, 37), psoriasis and atopic eczema (38), osteoarthritis (39), and human atherosclerotic tissue (40), many of which are characterized by infiltration of immunocytes including macrophages, DCs, and T/B-cells. One can conclude from these studies and studies of miR-21 expression in cancerous tissue that increased miR-21 may act as a general biomarker of diseased tissue and in particular inflammation-associated diseases.

In a similar fashion, many studies of circulating miRNA profiles have implicated miR-21 as a secreted biomarker of disease due to its association with exosomes – small cell-derived vesicles whose cargo contains stable small RNAs including miRNA (41). Exosomes have been implicated as a mechanism of cell-to-cell communication and in this way act as classic immunomodulators. The fact that miR-21 is found in many exosomes including tumor-derived and immunocyte-derived (42, 43) supports a role for miR-21 as a key modulator of immune processes.

Few studies thus far have implicated the specific cell type responsible for the increased miR-21 expression *in vivo*. However, a recent study of miR-21 expression in gastric cancer found increased stromal, but not tumor cell, miR-21 to be strongly linked to clinical–pathological features of disease (44). *In vivo* analysis of mice challenged with *Aspergillus fumigatus* to model airway inflammation showed that miR-21 was induced in cells of the monocyte/macrophage lineage (26).

### Inferring function from expression/induction studies

**Figure 1:**
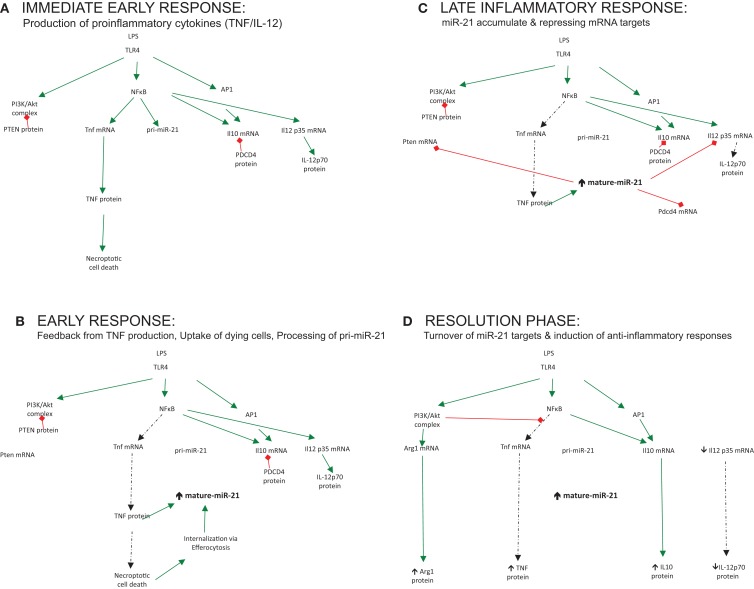
**The role played by miR-21 regulating output of immune responses over time**. **(A)** Immediate early response: production of proinflammatory cytokines (TNF/IL-12). **(B)** Early response: feedback from TNF production, uptake of dying cells, processing of pri-miR-21. **(C)** Late inflammatory response: miR-21 accumulate and repressing mRNA targets. **(D)** Resolution phase: turnover of miR-21 targets and induction of anti-inflammatory responses.

miR-21 is induced by many pro-inflammatory stimuli, both PAMPs and DAMPs, which trigger the inflammatory circuit and power up the cells of the immune system for action, illustrated in Figure 1A (immediate early response). However, the question remains as to what exact processes in this circuit this induced miR-21 regulates. The delayed induction of miR-21 in inflammatory reactions suggests that miR-21 may in fact negatively regulate the process of inflammation and be an important switch for the resolution of inflammation and maintenance of homeostasis, in essence counteracting the circuit, functioning as a trip-switch to turn off the often-damaging excessive pro-inflammatory response.

## “Making the Switch” – Feedback of miR-21 as a Novel Regulator of Inflammatory Responses

The notion that miR-21 serves to limit inflammation and promote resolution should be supported by profiling studies of macrophage subsets; however, little induction of miR-21 is seen in alternatively-activated macrophages treated with IL-4 or IL-10 alone (45, 46). This supports the theory that an initial damage or danger signal needs to occur, which promotes an early pro-inflammatory stimulus such as NFκB or AP-1 to trigger miR-21. This ensures that miR-21 induction is appropriately activated to counteract damage triggered by infection. Recently, enhanced miR-21 expression was reported when LPS-treated macrophages were treated with apoptotic cells (47). This event fueled miR-21 expression to a greater extent, which was associated with decreased pro-inflammatory responses and the resolution of inflammation. Additionally, in an *in vivo* murine model of peritonitis, high level of miR-21 was reported, which was increased further following treatment with Resolvin D1, a lipid mediator that promotes resolution of inflammation (48). In this respect, damage or infection can be seen as the fuel that fires miR-21 expression and only when these have been appropriately sensed will miR-21 be appropriately up-regulated to counteract this, illustrated in Figure 1B (early response). This control mechanism ensures that miR-21 and its associated processes are not wastefully induced but “switched on” at appropriate times – when required to change the direction of the circuit and affect the balance of the inflammatory reaction to promote healing, resolution, and a return to homeostasis.

Ultimately, however, the function of a particular miRNA cannot be solely inferred from studies of its induction but must be deduced through studies of its activity also – namely, the specific mRNA targets it represses in any given context. For many miRNAs, bioinformatics analysis has aided the prediction and discovery of relevant mRNA targets. This computational-based approach has been less successful for miR-21, with many possible mRNA targets verified through various innovative techniques in cancer, inflammation, and other contexts, all of which will not be discussed here. Instead, I will limit the discussion to those identified targets and processes affected by miR-21, which tell us most about its role in immune responses (illustrated in Figure 1C – late inflammatory response) and discuss how these may promote the negative regulation of the inflammatory circuit, illustrated in Figure 1D (resolution phase).

### PDCD4 and cytokine production

Our initial studies manipulating miR-21 during LPS signaling found that it had a unique effect on the levels of the anti-inflammatory cytokine IL-10, not observed for other cytokines. This was linked to the regulation of a proposed negative regulator of IL-10 production, PDCD4, loss of which was shown to protect from LPS lethality (25). Although the mechanism whereby PDCD4 regulates IL-10 and other cytokines remain an area of active investigation (49, 50), other groups have demonstrated that the miR-21/PDCD4 axis represents a key target for immunoregulation in multiple contexts, namely in protecting from type 1 diabetes (51), as a target for the endogenous danger ligand decorin-1 (52) and in regulating T-cell activation and polarization in SLE (53).

Recently the miR-21/PDCD4 axis was shown to play a key role in the process of efferocytosis (47) – the digestion and elimination of dead or dying cells by phagocytes, including macrophages, often associated with the induction of anti-inflammatory “clean-up” genes such as IL-10. Das et al. demonstrate that miR-21 levels are enhanced further in LPS-activated macrophages due to the uptake and internalization of apoptotic cells and, importantly, this process regulates IL-10 induction through PDCD4.

### PTEN/PI3K signaling

This study by Das et al. also clearly demonstrates a role for miR-21 in the regulation of TNF production, which separately from the miR-21/PDCD4 axis, is regulated by an additional miR-21 target gene, PTEN (47). A key intracellular kinase, PTEN is an important regulator of the PI3K/Akt pathway, which functions in many different cell types, each with unique functions and outcomes, but most strongly being pro-survival (54). It is not surprising then that elimination of PTEN, a negative regulator of PI3K activity, by dysregulated miR-21 would promote growth and survival in dividing somatic cells leading to malignant transformation (15, 55).

PTEN and PI3K signaling pathways have also been recently linked to macrophage phenotype and differentiation of functional subsets. Recently, studies of PTEN-deficient animals show more alternatively-activated macrophages in various models of polarization including Kuppfer cells, serving to protect from liver ischemia–reperfusion injury (56), as well as peritoneal macrophages marker expression (56, 57). The classical M2 marker Arg1, which is a key target for PI3K/Akt1 signaling, was found at much higher levels in these cells (57). Sahin et al. went on to demonstrate that this increased Arg1 expression resulted from activation of CEBPβ and STAT-3, as well as negative regulation of NFκB activity. Thus, miR-21 induction forms part of a key feedback circuit to limit excessive NFκB activity, turn off TNF production, and thereby transform the activated macrophage into a more reparative, “clean-up” cell, with key processes such as efferocytosis of dying cells, enhancing and promoting induction of this important immune modulator.

### TNF production

TNF has been associated with cell death and more recently high levels of TNF have been implicated in the death process observed in inflammatory macrophages labeled “necropoptosis” (58). Negative regulation of TNF levels by miR-21 therefore may not just help dampen down excessive inflammation but may also explain the effects of miR-21 on cell proliferation, migration, invasion, and transformation associated with excessive miR-21 levels and cancer. Strikingly, reciprocal regulation of miR-21 and TNF may in fact constitute an autoregulatory loop with evidence accumulating that TNF can promote miR-21 biogenesis (32) as well as the turnover of PDCD4 in macrophages (50). Moreover, the switch toward an anti-inflammatory, M2-like phenotype, and general immuno-regulatory environment, characterized by elevated IL-10 protein and increased Arg1 macrophage expression consistent with decreased TNF, which is associated with increased miR-21 (illustrated in Figure 1), may also account for poor immune responses against tumor-cells characteristic of tumor-associated macrophages (TAMs) found in cancer-induced stroma. Thus, stromal miR-21 induction (44) may constitute a pathogenic step in the biogenesis of cancer that leads to associated immunosuppression facilitating tumor growth and dissemination.

### IL-12 and antigen-presenting cells

The recent description of miR-21-deficient animals with profound defects in Th2 responses and skewing toward a Th1 response following administration of the OVA antigen (59) not only confirms the importance of miR-21 in directing T-cell polarization through its effects on innate antigen-presenting cells but also identifies T-cells and the adaptive immune response as a key target of miR-21 activity in immunity. Importantly, this study builds on earlier work (26) confirming an important target mRNA for miR-21; the IL-12p35 mRNA. IL-12, which acts as a strong inducer of Th1 responses and drives IFNγ production, is tightly controlled and in fact the p35 subunit is found at much lower levels in DCs and macrophages than its IL-12p70 partner, p40 (60). The finding that miR-21 can fine-tune its expression with big effects on subsequent immune responses *in vivo*, highlights the importance of this tiny 7 nt base-pair interaction. By directing the development of an appropriate T-cell response, this interaction again supports the notion that miR-21 controls the balance of pro- and anti-inflammatory responses. Accordingly, in diseases where miR-21 expression is dysregulated, this balance is altered with subsequent effects on innate but also adaptive immune cells, resulting in pathogenesis.

## Impact of Dysregulated miR-21 on Immune Responses

### Inflammatory diseases

If miR-21 does indeed represent a key switch in the transition from a pro-inflammatory to an anti-inflammatory response, it stands to reason that at times, this key control point will become dysregulated with impact on the overall immune response, altering the control and balance of the whole circuit, which manifests as disease. As mentioned above, elevated miR-21 has been reported in many disease states. On the one hand, increased miR-21 expression is associated with conditions characterized by impaired immune responses including asthma (26), psoriasis (38), cancer (5), and importantly chronic bacterial or viral infections (61–66) (discussed below). Many of these conditions are associated with reprograming of pro-inflammatory M1 macrophages and/or Th1-cells and the appearance of regulatory immune cells including M2 macrophages, Th2, or regulatory T-cells. Therefore, miR-21 dysregulation by different triggers (DAMPs or PAMPs) may in fact promote disease pathogenesis by promoting an anti-inflammatory, immunosuppressive environment.

Conversely, increased miR-21 expression has also been reported in diseases fueled by chronic inflammation including colitis (67), atherosclerosis (40), type 2 diabetes (68), and SLE (53). In these cases, triggering a regulatory response through miR-21 would be beneficial, yet this is not manifested in the inflammatory environment of these diseased tissues. miR-21 up-regulation may simply be a marker of increased inflammation in these tissues, induced by the “fire” of the pro-inflammatory milieu. Curiously, ablation of miR-21 in some of these models, including colitis (67) and psoriasis (69), has actually been shown to offer protection from disease, indicating that miR-21 activity is promoting inflammation in these cases. In some cancer models, miR-21 expression itself is associated with inflammatory activation. It can promote NFκB activation in breast cancer cells (70) and TNF and IFNγ production in activated T-cells (71). Here, miR-21 is clearly acting to induce inflammation in transformed tumor-cells and activated T-cells rather than suppress inflammation in infected or activated macrophages. Differences in its function may relate to the different target mRNAs engaged in each cell type. Alternatively, miR-21 may augment general inflammation – both pro- and anti-inflammatory (50), with the reported effects on macrophage output observed after miR-21 modulation simply being reflective of other cues/signals in these cells at the same time.

### Tumor promoting activity

Undoubtedly, miR-21 overexpression drives transformation of somatic cells and promotes tumorigenesis through effects on cellular growth, migration, and invasion (11–16). It is likely that the tumor microenvironment itself is also affected by miR-21 activity. As highlighted above, TAMs, which are re-programed from initial tumoricidal macrophages recruited to the site to immuno-permissive M2-like macrophages, are key cell types within the tumor microenvironment where miR-21 may be exerting pro-tumorigenic effects. Secretion of miR-21 from tumor-cell-derived exosomes or up-regulation of miR-21 in TAMs by tumor-derived pro-inflammatory products such as IL-6 or TNF, may participate in TAM reprograming and thereby facilitate growth, intravasation, and spread of tumor cells.

At the same time, as tumor-cells develop, intrinsic miR-21 may shape their responsiveness to therapy resulting in a more aggressive tumor phenotype. Alongside another immuno-responsive miRNA, miR-146, which functions as a negative regulator of TLR signaling pathways (72), miR-21 has been associated with chemoresistant ovarian epithelial cells (73). Importantly, these cells are characterized by low MyD88 expression and this key pro-inflammatory signaling protein has emerged as a key target for both miR-146 and miR-21. Low MyD88 expression in these aggressive cancer cells argues that TLR/IL-1 signaling may promote anti-cancer responses at this stage of disease and again dysregulation of miR-21 in the tumor acts to promote pathogenesis of disease progression.

### Infection

As miR-21 regulates immune responses, it stands to reason that its induction may represent a target for subversion by invading pathogens in the ever-evolving arms race between the mammalian immune system and microbes. Indeed, many studies have characterized the rapid induction of miR-21 following infection of macrophages and other cells with microbes, including the pioneering work by Cameron et al. This study demonstrated that EBV induces miR-21 during latency, linking this miRNA with viral persistence (61). In a similar manner, infection of hepatocytes with either HBV or HCV was recently shown to induce miR-21 (62, 65), and in addition to promoting viral replication by enhancing growth and survival of the infected cell, miR-21 induction also modulates the host response in favor of the virus. Interestingly, signaling components of the TLR system (MyD88 and IRAK) have emerged from these studies as targets for miR-21 with the downstream consequence of decreased induction of anti-viral interferon-α during infection (62). Similarly, infection of renal cells with pseudorabies virus (PRV) induces miR-21, which targets mRNA for the important host chemokine CXCL10/IP-10 (63).

Studies of pathogen-induced miR-21 not only tell us more about the important immune-relevant target mRNAs for miR-21 but also about immune evasion strategies employed by the pathogen. For example, mycobacterial species, which persist and replicate in macrophages by successfully interfering with host responses, have been shown to induce miR-21. This subsequently targets multiple components of key pathways required for mycobacterial containment, including vitamin-D-dependent induction of anti-microbial peptides and the induction of pro-inflammatory cytokines including IL-1, TNF, IL-12, and IFNγ (64, 66). In particular, the finding that the avirulent mycobacterial strain BCG, used with mixed success to vaccinate against tuberculosis worldwide, induces miR-21 to escape immune responses (66), supports the notion that blocking miR-21 may in fact boost immunity and therefore temporal and specific inhibition of miR-21 may be an ideal candidate for vaccine development.

## Rewiring the Circuit – miR-21 as an Attractive Target for Therapeutic Intervention?

With interest in antisense technology increasing due to improved delivery techniques, specific targeting and more effective chemistries emerging, programs to target miR-21 in disease are being developed (74). Published studies have shown beneficial effects in various models although the exact mechanism contributing to this remains unclear. These studies are listed in Table 2, which highlights differences and commonalities in the methodologies and approaches used. Although effects of silencing miR-21 using antisense technology to counteract cardiac fibroblast remodeling in response to stress (75) were not reproducible in miR-21-deficient mice (76), there remains interest in blocking miR-21’s pro-fibrogenic activity particularly in response to ischemic–reperfusion injury (77). Early studies using anti-miR technology to block interstitial fibrosis demonstrated that protection from disease was generated through modulation of the key metabolic sensor, and miR-21 target, PPARα (78). However, it remains possible that miR-21 can exert some of its pro-fibrogenic activities through regulation of inflammatory signaling pathways such as IL-10 and TGF-β. Antisense to miR-21 has also been shown to reduce disease in two models of chronic inflammatory disease – psoriasis and SLE, with miR-21 inhibition in these cases apparently reducing inflammation, through effects on T- and B-cell activation and proliferation (in the SLE model) (79) and through negative regulation of MMP activity and TNF production (in the inflamed epidermis in the psoriasis model) (69).

**Table 2:** **Published studies employing antisense to miR-21 to block disease**.

Disease model	Oligonucleotide technology	Treatment	Result	mRNA targets	Reference	Company
Cardiac hypertrophy	AntagomiR – cholesterol modified	Daily – 3 days, 80 mg/kg	Protection – less cardiac damage and fibrosis	SPRY	(75)	Alnylam Pharma
	Anti-miR – sugar modified phosphothiorate backbone	As above	Protection		(80)	Regulus Therapeutics
	LNA (8-mer)	As above	No difference		(76, 80)	

Renal fibrosis	Anti-miR−	Daily – 3 days 20 mg/kg	Protection – decreased interstitial fibrosis	PPARα	(78)	Regulus Therapeutics

SLE	Locked nucleic acid (LNA)	12 weeks (Prime + 3-weekly) 25 mg/kg	Protection – decreased splenomegaly	Not defined	(79)	Santaris Pharma

Psoriasis	LNA		Protection	TIMP3	(69)	Sataris Pharma

Recent basic science studies attempting to understand miR-21’s complex biology better, are affecting targeting strategies and the development of miR-21 modulators for disease. Intriguingly, a study of miR-21 overexpression in hepatocytes observed differences in mRNA target engagement dependent upon the degree of overexpression, correlating with dysregulation of miR-21 in diseased tissue (81). This confirms that miR-21 behaves differently under various circumstances, including the level of miR-21 up-regulation itself and will affect strategies to target miR-21 in diseased tissue. With the widespread availability of advanced transcriptomic technologies, we may need to move toward a closer examination of elevated miR-21 and its impact upon the host cell transcriptome to get a clearer picture of the exact processes regulated by this particular miRNA.

As with any therapy designed to alter the balance of immune responses, the possibility of off-target effects or predisposition to other conditions, perhaps those characterized by chronic inflammation, exists. With greater understanding of miR-21 regulation and function, we may be able to tailor RNA-therapies and avoid off-target consequences. Furthermore, the notion of targeting specific miRNA:mRNA interactions via morpholino technology may avoid the deleterious effects of broad inhibition of miR-21 (82) and more specific delivery technologies such as β-glucan microparticles, which hone specifically to macrophages could be utilized to limit the effects of inhibition to a specific target cell-type (83). The transience and high turnover of RNA itself may help limit the effects of antisense treatment to the short-term. At the same time, caution needs to be erred when inhibiting an miRNA as ubiquitous and promiscuous as miR-21. Indeed, the area of miR-21 turnover and decay itself remains an unexplored area and its study may enhance our understanding of the role of miR-21 in immune responses, providing alternative means to antisense technology for limiting its expression *in vivo*.

## Adaptive Immunity

Thus far, this review has concerned itself with miR-21 induction in cells of the innate immune system. However, as alluded to earlier, miR-21 is also found in both T and B-cells and its role in these cells is the subject of much investigation. Thus, employment of strategies to target miR-21 for modulation of immune responses requires anticipation of the effects on these cell types also.

Profiling studies of T-cells indicate that miR-21 is induced and acts as a marker of activated T-cells (19, 20), promoting survival and activation of these cells (84–86). In this way, miR-21 induction serves as a means to stratify naïve from activated T-cells, possibly assisting in the co-ordination of T-cell memory. Despite its expression across multiple T-cell subsets, intrinsic miR-21 can also affect T-cell polarization. Naïve T-cells transfected with miR-21 develop a more Th2/Treg phenotype (87) and this may be due to engaging different targets expressed in response to various other polarizing signals including BCL-6. T-cell miR-21 may also play an important role regulating tolerance to self, as demonstrated by studies showing exaggerated miR-21 induction in activated T-cells from PD1-deficient mice (88). These studies highlight the dual roles that T-cells play in regulating immune responses. While they must promote pro-inflammatory responses and eliminate infected cells, they must also orchestrate clearance of infection and promote resolution. T-cell miR-21 seems treads a fine line in balancing these processes and may become dysregulated during cases of autoimmunity.

## Conclusion

Over the last 10 years, much effort has been placed in profiling the miR-nome of various cells under different conditions. From this, miR-21 has emerged as important miRNA both highly expressed and dynamically regulated in various cell types. Since then, identification of miR-21 function has been complicated not only by the possibility for many mRNA target interactions but also by its complex regulation in response to extracellular signals. The possibility has emerged that miR-21 can regulate numerous processes involved in correct cell function, survival, and proliferation, which if interrupted, can predispose to cellular transformation. However, it has also been linked to key processes involved in inflammation, detecting and responding to disturbances in homeostasis throughout the body, and orchestrating these responses appropriately. miR-21 therefore plays a dynamic role in inflammatory responses. Unlike other mediators, its presence is not solely characteristic of a pro-inflammatory or an immunosuppressive state, but is acting as a key signal mediating the balance and transition between both states. In essence, miR-21 induction can be seen as a “molecular rheostat” regulating the inflammatory switch. This makes it a novel and attractive target for therapeutic intervention and enhanced knowledge of its specific mRNA targets, as well as the signaling pathways and cellular processes regulated by miR-21, can only enhance its usefulness and attractiveness in this area.

## Conflict of Interest Statement

The author declares that the research was conducted in the absence of any commercial or financial relationships that could be construed as a potential conflict of interest.
